# An Integrative Study on the Inhibition of Bone Loss via Osteo-F Based on Network Pharmacology, Experimental Verification, and Clinical Trials in Postmenopausal Women

**DOI:** 10.3390/cells12151992

**Published:** 2023-08-03

**Authors:** Mi Hye Kim, Minkyung Bok, Hyunjung Lim, Woong Mo Yang

**Affiliations:** 1 Department of Convergence Korean Medical Science, College of Korean Medicine, Kyung Hee University, Seoul 02447, Republic of Korea; kimmihye526@khu.ac.kr; 2 Department of Medical Nutrition, Graduate School of East–West Medical Science, Kyung Hee University, Yongin 17104, Republic of Korea; yoccobbong03@naver.com; 3 Research Institute of Medical Nutrition, Kyung Hee University, Seoul 02447, Republic of Korea

**Keywords:** bone anabolic effect, clinical trials, network analysis, Osteo-F, osteoporosis

## Abstract

The inhibition of bone loss remains a challenge for postmenopausal women, considering the fact that only three anabolic treatments for osteoporosis have been approved by the FDA. This study aimed to investigate the osteogenic capacities of Osteo-F, a newly developed herbal formula, upon integrating network analysis and pre-clinical studies into clinical trials. The network pharmacology analysis showed that a potential mechanism of Osteo-F is closely related to osteoblast differentiation. Consistent with the predicted mechanism, Osteo-F treatment significantly enhanced bone matrix formation and mineralization with collagen expression in osteoblasts. Simultaneously, secreted bone-forming molecules were upregulated by Osteo-F. After the administration of Osteo-F to osteoporotic mice, the femoral BMD and osteocalcin in the serum and bone tissues were significantly improved. Subsequently, a randomized, double-blinded, placebo-controlled clinical trial showed that 253 mg of Osteo-F supplementation for 24 weeks resulted in significant improvements in the Z-score and serum osteocalcin levels of postmenopausal women compared to the placebo, thus indicating bone anabolic efficacy. In the current study, the bone anabolic effect of Osteo-F was determined by activating the differentiation and mineralization of osteoblasts through integrating experiments based on network analysis into clinical trials, with synchronized, reliable evidence, demonstrating that Osteo-F is a novel bone anabolic treatment in postmenopausal women.

## 1. Introduction

Approved therapies for osteoporosis include osteoanabolic therapies that promote osteoblast activity and anti-resorptive therapies that inhibit osteoclast activity [1]. An imbalance between bone formation and bone resorption leads to a reduction in bone mass and mechanical strength [2]. The most widely used treatments focus on anti-resorptive medications targeting osteoclast-mediated bone resorption, thereby attenuating bone loss and increasing bone mineral density (BMD) [3]. Considering bone resorption treatments, the speed of development is relatively slow for bone-forming medications that induce the production of osteoblast-derived bone tissue [4]. Given the evidence that practitioners are currently discouraged from prescribing the long-term use of anti-resorptive osteoporotic drugs, such as bisphosphonates and denosumab, because of their side effects, including brittleness, microdamage accumulation, and bone mineral loss rebound, the search for new therapeutics for the management of bone loss with an underlying mechanism of bone formation is important to advance the treatment of osteoporosis [5]. 

Typical osteoanabolic medications, such as teriparatide and romosozumab, were reported to reduce nonvertebral and vertebral fractures more effectively and faster than anti-resorptive medications [6]. Approximately 100 million people worldwide who are affected by this enfeebling osteoporotic disease need bone-forming agents, which, in turn, lead to fracture risk reduction without any unwanted adverse effects [7].

Osteo-F, a new herbal formulation for improving bone loss, consisting of the fruits of *Schizandra chinensis* (Turcz.) Baill. (Magnoliaceae) and *Lycium chinense* Mill. (Solanceae), as well as the root of *Eucommia ulmoides* Oliv. (Eucommiaceae), was suggested to alleviate bone loss in osteoporotic mice as an osteoanabolic agent based on our previous studies [8,9]. It increased bone-forming molecules, including bone morphogenetic protein (BMP)-2, osteopontin (OPN), runt-related transcription factor 2 (Runx2), and Osterix [8,9]. This study aimed to reveal the mechanism of action of Osteo-F in silico and verify its ameliorative effects on osteoporosis through in vitro and in vivo experiments. Today, network pharmacology, a new computational tool used to examine network connectivity, is performed by establishing a “compound–protein/gene–disease” network [10,11]. The identification of biological and pharmacological targets from the compounds of medicinal herbs through network pharmacology analysis has emerged as the next paradigm in the field of drug discovery and development [12]. To this end, we undertook a drug development study that predicted the potential target, constructing the “network-target, multiple-component-therapeutics”, and evaluated the therapeutic actions of traditional medicines in pre-clinical studies, both in vitro and in vivo, increasing the success rate of clinical trials. On the basis of these results, we aimed to provide the clinical safety and efficacy of Osteo-F in terms of bone health among Korean postmenopausal women in a 24-week, randomized, double-blinded, and placebo-controlled trial.

## 2. Materials and Methods

### 2.1. Gene Set Construction and Network Analysis

The components of *Schizandra chinensis*, *Lycium chinense*, and *Eucommia ulmoides*, of which Osteo-F consists, were collected based on TM-MC. After removing duplicates and compounds with no target information, 173, 78, and 45 compounds were culled for targets of *S*. *chinensis*, *L*. *chinense*, and *E*. *ulmoides*, respectively. Afterward, genes with co-occurrence in the literature on compounds were gathered through PubChem. For each herb, 926, 828, and 568 genes were determined to be related genes through the STRING database (http://www.string-db.org/, accessed on 17 March 2021) with a score ≥ 0.7, which represents high confidence (Table S1). After eliminating the common elements, the whole gene set of Osteo-F included 1470 target genes (Table S2). For the gene set of osteoporosis, osteoporosis-related genes were collected through GeneCards (https://www.genecards.org/, accessed on 17 March 2021), MalaCards (https://www.malacards.org/, accessed on 17 March 2021), and DisGeNet (https://www.disgenet.org/, accessed on 17 March 2021), and 4273, 290, and 1098 target genes were gathered, respectively (Table S3). After removing common genes, in total, 4582 genes were used to build the gene set of osteoporosis (Table S4).

### 2.2. Preparation of Osteo-F

All Osteo-F samples were provided by BOIN BIO Convergence Co., Ltd. (Seoul, Republic of Korea). Osteo-F was extracted following the procedure of Lee et al. [8]. Briefly, 200 g of dried fruits of *Schizandra chinensis*, 100 g of dried fruits of *Lycium chinense*, and dried radix of *Eucommia ulmoides* were extracted in 10× distilled water at room temperature and 60 °C for 24 h, respectively. The respective extracts were filtrated with a 1 μm filter paper and combined. All extracts were concentrated using a rotary evaporator under decompression by 65 brix% and powdered by a freezing dryer (yield: 57%). The powder was made from the Osteo-F tablet to facilitate administration, transportation, and storage.

### 2.3. Mineralized Matrix Formation Assay

Human osteoblast-like cell line SaOS-2 cells supplemented with Dulbecco’s modified minimal essential medium (DMEM; Gibco, Grand Island, NY, USA), 10% fetal bovine serum, and 1% penicillin were seeded in 6-well plates at a density of 0.8 × 10^5^ cells/well in an atmosphere of 5% CO_2_. The cells were incubated in an osteogenic culture medium including 50 μg/mL L-ascorbic acid (AA; Fisher Scientific, Oxford, UK) and 10 mM β-glycerophosphate (β-GP; Sigma-Aldrich Inc., St. Louis, MO, USA) to induce osteoblast differentiation. Osteo-F at the concentrations of 1, 10, and 100 μg/mL was treated through whole experiments. After 10 days, the cell fixation was performed in 10% neutralized formalin and 0.1% Triton X-100. The cells were stained with the 40 mM Alizarin Red-S (pH 4.2) (Sigma-Aldrich Inc., St. Louis, MO, USA) and monitored under a microscope using the Leica Application Suite (LAS; Leica Microsystems, Buffalo Grove, IL, USA). To quantify the amount of differentiation ratio, the supernatant was discarded, and a solution containing 20% methanol and 10% acetic acid was added to cells for 2 h. The incubated medium was read at 570 nm using an enzyme-linked immunosorbent assay (ELISA) reader (Molecular Devices, Downingtown, PA, USA).

### 2.4. Bone Formation-Related Markers Content by Reverse Transcription-Polymerase Chain Reaction (RT-PCR)

After differentiation for 10 days of SaOS-2 cells, the TRIzol method was used to isolate the RNA from cells. RNAs were treated with a complementary DNA kit (Maxime RT premix kit; Invitrogen, Carlsbad, CA, USA) and verified for subsequent reverse transcription in order to evaluate the expression of the following selected genes of interest, respectively: GAPDH, 5′-GGCATGGACTGTGGTCATGA-3′ and 5′-TTCACCACCATGGAGAAGGC-3′; COL, 5′-TAGGCCATTGTGTATGCAGC-3′ and 5′-ACATGTTCAGCTTTGTGGACC-3′; OCN, 5′-AGCTCAACCCCAATTGTGAC-3′ and 5′-AGCTGTGCCGTCCATACTTT-3′; BSP-1, 5′-GAGCCAGGACTGCCGAAAGGAA-3′ and 5′-CCGTTGTCTCCTCCGCTGCTGC-3′; BMP-2, 5′-GCGGTGGACTGCACAGGGAC-3′ and 5′-CTACCCTTCCCCGTGGGGGA-3′; ALP, 5′-TGGAGCTTCAGAAGCTCAACACCA-3′ and 5′-ATCTCGTTGTCTGAGTACCAGTCC-3′.

### 2.5. Bone Formation-Related Markers Content by Western Blotting Analysis

Proteins of the differentiated SaOS-2 cells were extracted with a RIPA buffer (Tech & Innovation, Gangwon, Republic of Korea) containing protease inhibitors (Roche, Basel, Swiss) and separated on sodium dodecyl sulfate-polyacrylamide gel electrophoresis. The isolated proteins were transferred onto a membrane (Bio-Rad, Hercules, CA, USA) and incubated with mouse and rabbit monoclonal anti-OCN, -RUNX family transcription factor 2 (Runx2), -Osterix and -ALP (Cell Signaling Technology; Danvers, MA, USA). After incubation with anti-mouse and rabbit IgG secondary antibody conjugated with horseradish peroxidase (Cell Signaling Technology), the immunoreactive bands were visualized by a chemiluminescence imaging system (GE Healthcare, Little Chalfont, UK) using enhanced chemiluminescence reagents. The band intensity was quantified using the Image J software (v1.4.3.x., U.S. National Institutes of Health, Bethesda, MD, USA). All experiments were performed in triplicate.

### 2.6. Animal Experiment

The animal studies were performed with five-week-old female ICR mice (weight 20–22 g) obtained from Raonbio Inc. (Yongin, Republic of Korea). All experiments were performed according to the guidelines of the Guide for the Care and Use of Laboratory Animals of the National Institutes of Health and approved by the Committee on the Care and Use of Laboratory Animals of Kyung Hee University (KHUASP(SE)-18-071). Mice were grown in the well-controlled room, maintaining a 12 h light/dark cycle at 20 ± 5 °C and 55 ± 15% humidity. Tap water and a standard chow diet were provided to all mice. After 1 week of adaptation, all mice (seven mice per group) were under surgery. After recovery and induction for 8 weeks, 17β-estradiol (E2) and Osteo-F were treated intraperitoneally and orally, respectively, for 8 weeks. Mice were assigned into six groups: (1) Sham, sham-operated normal control group, (2) OVX, ovariectomized negative control group, (3) E2, 10 μg/kg of E2-injected OVX positive control group, (4) Osteo-F 0.52 (0.52 mg/kg of Osteo-F-administrated OVX group), (5) Osteo-F 5.2 (5.2 mg/kg of Osteo-F-administrated OVX group), and (6) Osteo-F 52 (52 mg/kg of Osteo-F-administrated OVX group). All samples, including vehicle, E2, and Osteo-F, were treated once a day for 5 days per week. The administration dose of Osteo-F in mice was decided based on the human equivalent dose equation. After a total of 16 weeks of experimentation, 8 weeks for induction of osteoporosis, and 8 weeks for treatment, all mice were sacrificed.

### 2.7. Bone Mineral Density Determined by Dual Energy X-ray Absorptiometry

The distal femur was detached and the bone mineral density (BMD) was analyzed using dual-energy X-ray absorptiometry (DXA; Medikors Inc., Seongnam, Republic of Korea). The value was indicated by g/cm^2^ for BMD.

### 2.8. Osteocalcin Concentration in Serum

The blood of mice was obtained by the orbital sinus method under anesthesia. The blood samples were centrifugated, and serum was collected. The concentration of serum OCN was estimated by an enzyme-linked immunosorbent assay (ELISA) kit (Immutopics, Inc., San Clemente, CA, USA). The procedures were conducted according to the manufacturer’s instructions.

### 2.9. Osteocalcin^+^ Expression in Femoral Bone Tissues by Immunofluorescence

The detached femoral bone tissues were fixed with 10% neutralized formalin for 24 h. After decalcification and 1 M ethylenediaminetetraacetic acid for 2 weeks, the tissue paraffin block was made for immunofluorescence. The bone tissues were sliced into 7 μm thickness and incubated with primary anti-rabbit OCN overnight at 4 °C. After washing, the samples were incubated with the anti-fluorescence quencher conjugated goat anti-rabbit OC for 2 h and then Topro 3 for counterstaining of nucleus for 3 min, respectively. Sections were observed using a fluorescence microscope (LSM 5 PASCAL; Carl Zeiss, Oberkochen, Germany).

### 2.10. Study Design for Randomized, Double-Blind, Placebo-Controlled Clinical Trial 

This study was a randomized, double-blind, placebo-controlled clinical trial for 24 weeks. The protocol was approved by the Kyung Hee University Hospital Institutional Review Board (IRB) (No: KMC IRB 2018-06-055). Women were eligible for the study if they were 45 or older, had more than a year since the last menstruation period, had both ovaries removed, were not taking menopause-related drugs or estrogen, and provided written informed consent for this trial. While patients were diagnosed with osteoporosis or under treatment, those who had diseases affecting osteoporosis (hypothyroid or parathyroid disease, Cushing syndrome, diabetes, kidney disease, ovarian cancer, and others) who had received hormone replacement therapy within the last three months, and who had undergone treatment for the spine were excluded. The study also excluded uncontrolled hypertension patients, body mass index (BMI) less than 18.5 kg/m^2^ or greater than 30 kg/m², kidney and liver function disorders, and those with other diseases that the researchers believed inappropriate for the test. The subjects were recruited from October 2018 to July 2019. A total of 132 subjects participated, 12 subjects were excluded due to violation of the criteria for exclusion, and 120 subjects were registered. Ten subjects withdrew consent, and twenty-eight subjects due to diseases were excluded during the experiment. The final analysis included 39 subjects in the test group and 43 subjects in the control group, except for dropouts.

Both the test product and the control product are 500 mg per tablet (once a day). The test product components are the compound extract (containing Osteo-F freeze-dried powder 253 mg, 50.6%), and the other ingredients are dextrin (19.8%), microcrystalline cellulose (28.5%), HPMC (1.0%), and caramel pigments (0.1%). The control product consists of only the same subsidiary material (dextrin (40.0%), microcrystalline cellulose (58.9%), HPMC (1.0%), and caramel pigments (0.1%)) as the test product without Osteo-F.

### 2.11. Measurement of BMD and Serum OCN, Ca, and PTH Levels in Humans 

BMD was measured at baseline and after 24 weeks in the lumbar spine L1-L4 and left femoral total using a DXA (BHR-100-P IDXA, GE LUNAR, Madison, WI, USA). Bone health-related biomarkers such as OCN, Ca, and PTH were measured at baseline and 24 weeks. Blood pressure, pulse, and adverse events were measured and recorded at each visit to evaluate safety.

### 2.12. Statistical Analysis

Significance was determined by one-way analysis of variance (ANOVA) and Tukey multiple comparison tests using SPSS Data Analysis Version 17.0 (SPSS Inc., Chicago, IL, USA). In all analyses, *p* < 0.05 was taken to indicate statistical significance. The clinical trial results were analyzed using SAS ver 9.4 (SAS Institute, Cary, NC, USA) for those who conformed to a certain level and completed the following protocol. The difference between the control and Osteo-F intake groups in efficacy endpoints was analyzed as a Student’s *t*-test. It was also analyzed as a paired *t*-test to compare the changes before and after in all counties. The safety analysis was compared to those who had the results of vital signs, physical examinations, and adverse effects before and after the clinical experiment.

## 3. Results

### 3.1. Gene Comparison between Osteo-F and Osteoporosis

**Figure 1:**
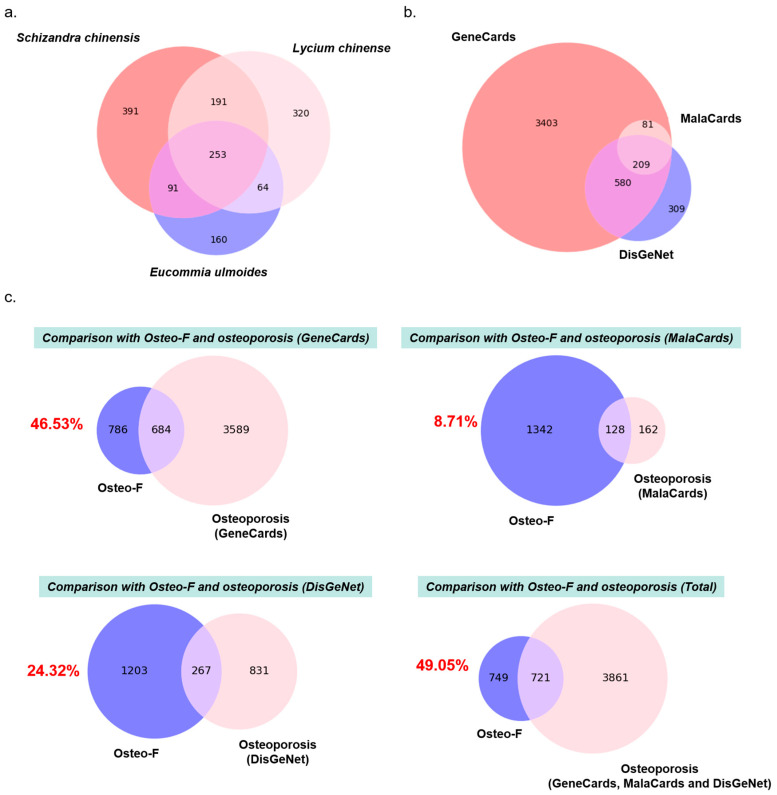
An integrated network analysis of Osteo-F. (**a**) Veen diagram showing overlapped genes between *Schizandra chinensis*, *Lycium chinense*, and *Eucommia ulmoides*. (**b**) Veen diagram showing overlapped genes of osteoporosis derived from GeneCards, MalaCards, and the DisGeNet database. (**c**) A comparison between Osteo-F and osteoporosis. Veen diagram of intersection targets between the Osteo-F network and the gene sets of osteoporosis.

The target genes of the components of *S. chinensis*, *L. chinense*, and *E. ulmoides* were 926, 828, and 568 genes, respectively (Figure 1a). The whole gene set of Osteo-F, including *S. chinensis*, *L. chinense*, and *E. ulmoides* was constructed with 1470 target genes. In the case of the osteoporosis gene set, 4273, 290, and 1098 target genes were collected from GeneCards, MalaCards, and the DisGeNet open database for a total of 4582 genes (Figure 1b). The gene sets of Osteo-F and osteoporosis were compared to examine the correlation between Osteo-F and osteoporosis. For three different gene sets of osteoporosis, which were based on GeneCards, MalaCards, and DisGeNet databases, compared to the gene set of Osteo-F, there were 684, 128, and 267 common genes accordingly. Summing the three osteoporosis gene sets, an osteoporosis gene set with 4582 genes was taken to investigate the correlation between Osteo-F and osteoporosis. Among the 1470 genes of Osteo-F and 4582 genes of osteoporosis, 721 genes were found in common, which indicates that 49.05% of the genes of Osteo-F were correlated to osteoporosis (Figure 1c).

### 3.2. Functional Enrichment Analysis of the Osteo-F Network

**Figure 2:**
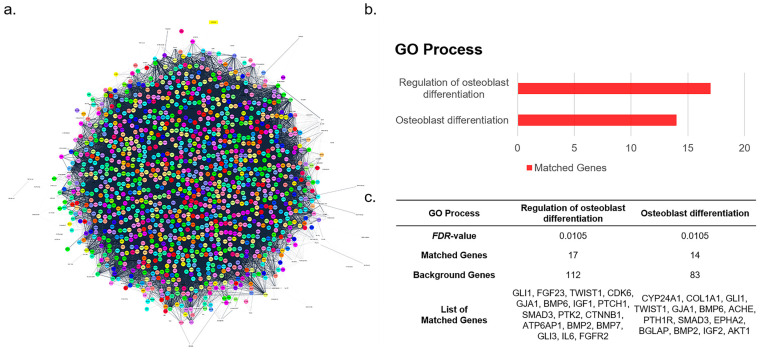
Functional target pathway of Osteo-F. (**a**) A network of Osteo-F with 1470 target genes derived from components of *Schizandra chinensis*, *Lycium chinense*, and *Eucommia ulmoides*. (**b**) Enrichment analysis extracted from the GO process of target genes of the Osteo-F network. (**c**) Detailed information of GO biological terms of “regulation of osteoblast differentiation” and “osteoblast differentiation” for the Osteo-F network.

From the target genes of Osteo-F, including *S*. *chinensis*, *L*. *chinense*, and *E*. *ulmoides*, a network was constructed. The Osteo-F network consisted of 1470 nodes and 149,987 edges (Figure 2a). Functional enrichment analysis based on the GO process was performed on the network of Osteo-F in osteoporosis. From the analysis, “regulation of osteoblast differentiation” and “osteoblast differentiation” were derived to be a potential function of Osteo-F in osteoporosis (Figure 2b). “Regulation of osteoblast differentiation” showed a 0.0105 FDR-value with 17 matched genes with Osteo-F, which were *GLI1*, *FGF23*, *TWIST1*, *CDK6*, *GJA1*, *BMP6*, *IGF1*, *PTCH1*, *SMAD3*, *PTK2*, *CTNNB1*, *ATP6AP1*, *BMP2*, *BMP7*, *GLI3*, *IL6*, and *FGFR2*. “Osteoblast differentiation” had an FDR value of 0.0105 with 14 matched genes with Osteo-F, which were *CYP24A1*, *COL1A1*, *GLI1*, *TWIST1*, *GJA1*, *BMP6*, *ACHE*, *PTH1R*, *SMAD3*, *EPHA2*, *BGLAP*, *BMP2*, *IGF2*, and *AKT1*. Among the 1470 genes for the Osteo-F-targeted genes, 15.18% (17/112) and 16.87% (14/83) matching rates were predicted with each of the functional biologic terms for the GO process “regulation of osteoblast differentiation” and “osteoblast differentiation” (Figure 2c).

### 3.3. Osteogenic Potential of Osteo-F in Mineralized Osteoblasts 

**Figure 3:**
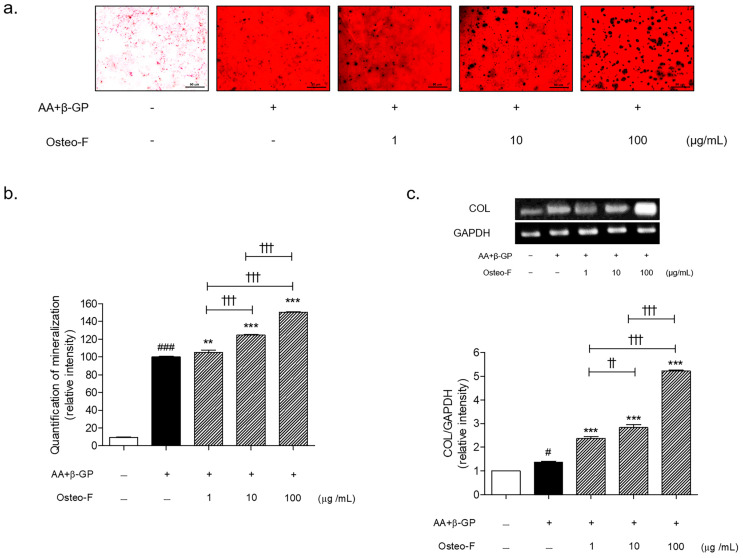
Osteogenic properties of Osteo-F in osteoblasts. (**a**) Morphological changes of mineralized SaOS-2 osteoblast cells under microscope with 400× magnification. (**b**) The red mineralized intensity of ARS staining. (**c**) Expression of COL in differentiated SaOS-2 osteoblast cells. Results are presented as mean ± standard error of the mean; ^#^ *p* < 0.05 and ^###^ *p* < 0.001 compared to non-treated cells; ** *p* < 0.01 and *** *p* < 0.001 compared to AA + β-GP-treated mineralized cells; ^††^ *p* < 0.01 and ^†††^ *p* < 0.001 compared to Osteo-F-treated mineralized cells.

Mineralized nodules stained by Alizarin Red S were significantly increased after incubation with a mineral induction medium for 10 days. Treatment with Osteo-F resulted in a dose-dependent increase in the osteoblast mineralized red intensity (Figure 3a,b). The calcium (Ca) nodules stained by black color were markedly increased in the Osteo-F-treated cells. The increase rates for Osteo-F treatment at 1, 10, and 100 μg/mL of mineralization were 5.2%, 24.7%, and 50.2%, respectively. The content of COL in the mineralized SaOS-2 osteoblasts was significantly increased by the Osteo-F treatment of 1, 10, and 100 μg/mL, which was similar to the result from the mineralization intensity (Figure 3c).

### 3.4. Increase of Bone Formation-Related Markers Expressions by Osteo-F in Mineralized Osteoblasts

**Figure 4:**
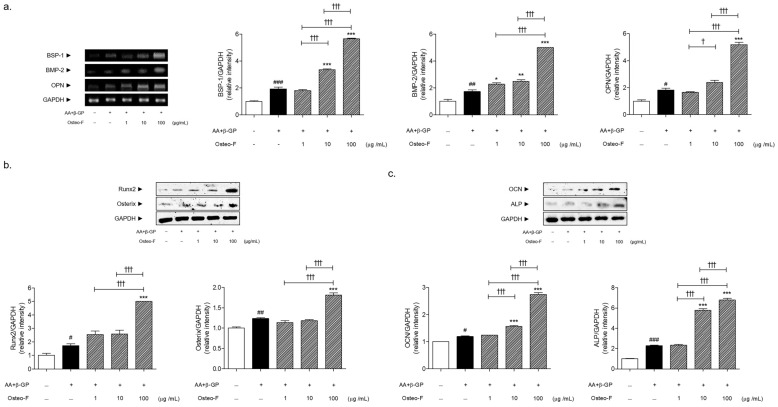
(**a**) Expression of BSP-1, BMP-2, and OPN mRNA in differentiated SaOS-2 osteoblast cells. (**b**) Expression of Runx2 and Osterix protein in differentiated SaOS-2 osteoblast cells. (**c**) Expression of OCN and ALP protein in differentiated SaOS-2 osteoblast cells. Results are presented as mean ± standard error of the mean; ^#^ *p* < 0.05, ^##^ *p* < 0.01 and ^###^ *p* < 0.001 compared to non-treated cells; * *p* < 0.05, ** *p* < 0.01 and *** *p* < 0.001 compared to AA + β-GP-treated mineralized cells; ^†^ *p* < 0.05 and ^†††^ *p* < 0.001 compared to Osteo-F-treated mineralized cells.

Cells treated with a differentiation medium exhibited a significant increase in OC protein expression when compared with non-treated cells. Following co-treatment with 1, 10, and 100 μg/mL of Osteo-F in the presence of a differentiation medium, the expressions of OCs were markedly increased by 4.57%, 31.35%, and 130.69%, respectively, compared to non-treated mineralized cells (Figure 4a). 

Differentiation medium exposure of osteoblastic cells induced a significant increase of bone formation-related factors, including bone sialoprotein-1 (BSP-1), BMP-2, and OPN by 1.93-, 1.72-, and 1.82-fold, respectively. There was a dose-dependent change in the BSP-1, BMP-2, and OPN expressions by the Osteo-F treatment in the mineralized SaOS-2 osteoblast cells (Figure 4b). The expressions of the BSP-1 mRNA level were significantly increased by 1.74 and 2.93 times in the 10 and 100 μg/mL Osteo-F-treated cells, respectively. The levels of BMP-2 and OPN were significantly increased by 190.02% and 185.84%, respectively, by the Osteo-F treatment of 100 μg/mL.

Furthermore, the increment rates of Runx2, Osterix, and alkaline phosphatase (ALP) were 1.72, 1.24, and 2.29-fold in the differentiated and mineralized cells compared to the non-treated cells. The treatment of Osteo-F at the 100 μg/mL concentration significantly increased the 2.90 and 1.47-fold protein expressions of Runx2 and Osterix. The ALP expressions in the 10 and 100 μg/mL Osteo-F-treated cells were increased by 2.53 and 2.97 times compared to the non-treated mineralized cells (Figure 4c).

### 3.5. Recovery of the BMD Level by Osteo-F in OVX-Induced Osteoporotic Mice

**Figure 5:**
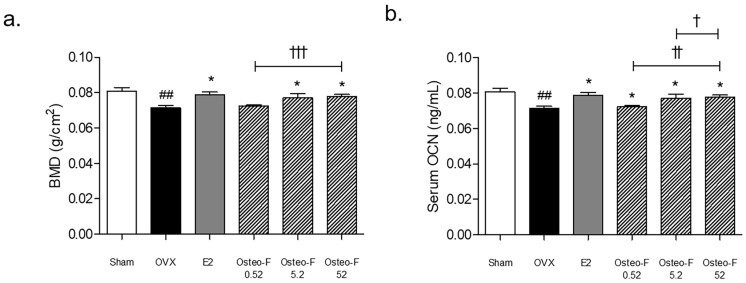
(**a**) Femoral BMD level in OVX-induced osteoporotic mice. (**b**) Serum OCN level in OVX-induced osteoporotic mice. Results are presented as mean ± standard error of the mean; ^##^ *p* < 0.01 compared to Sham group; * *p* < 0.05 compared to OVX-induced osteoporotic group; ^†^ *p* < 0.05, ^††^ *p* < 0.01 and ^†††^ *p* < 0.001 compared to Osteo-F-treated mineralized cells.

Compared to the sham group, a BMD loss in rats was observed in the ovariectomized (OVX) group. As shown in Figure 5a, the BMD levels of the femoral bone tissues were significantly increased in the OVX mice treated with 9 and 90 mg/kg of Osteo-F. 

### 3.6. Serum OCN and OCN^+^ Expression of Femoral Bone Tissues by Osteo-F in OVX-Induced Osteoporotic Mice

**Figure 6:**
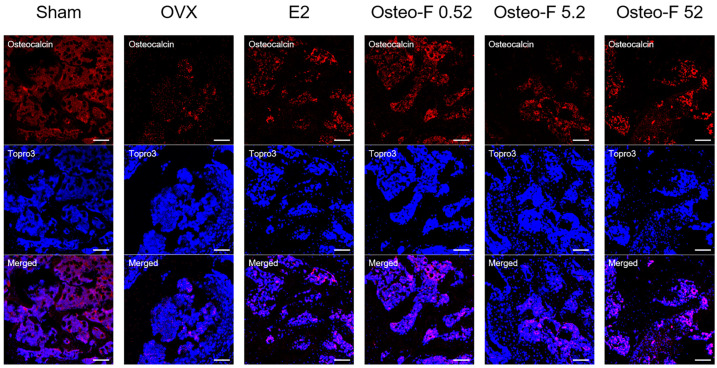
Immunofluorescence intensity of OCN expression in OVX-induced osteoporotic mice. Red, OCN; Blue, Topro3. The scale bar was 50 μm.

The serum osteocalcin (OCN) content in the OVX-induced osteoporotic mice was lower than that in the sham-operated mice, while the treatment of Osteo-F at 0.9, 9, and 90 mg/kg significantly upregulated the decline of the serum OCN concentrations in the OVX mice (Figure 5b). A similar trend was found in the femoral body of the bone tissues. The OCN^+^ expression was markedly decreased in the femur of OVX mice. Increasing expressions of OC^+^ intensities were observed in the femoral body of the 0.9, 9, and 90 mg/kg Osteo-F-treated groups (Figure 6).

### 3.7. Change in Biochemical Bone Markers including the Z-Score and T-Score by Osteo-F in Postmenopausal Women

Changes were observed in the bone mineral density and biochemical bone markers during 24 weeks in the treatment group. Among the bone markers measured by the DXA, lumbar spine BMD, bone mineral content (BMC), and T-score all tended to be increased in the Osteo-F group and decreased in the placebo group after 24 weeks from baseline, but no significant difference was observed. The lumbar spine Z-score showed a significant difference between the two groups (change: 0.07 ± 0.20 in the Osteo-F group vs. −0.02 ± 0.19 in the placebo group) when adjusting for age (*p* = 0.048). The T-score of the femoral total in the test group decreased after 24 weeks (−0.03 ± 0.09), showing a significant difference (*p* = 0.044). However, there were no significant differences in all femoral neck BMD, BMC, T-score, and Z-score values between the two groups after 24 weeks (Table 1).

### 3.8. Change in Serum Biomarkers including the PTH and PTH/Ca Ratio by Osteo-F in Postmenopausal Women

As a result of comparing the change in OCN after 24 weeks, the change in OCN in the test group was increased (0.79 ± 3.02 ng/mL), and that in the control group was decreased (−0.10 ± 3.86 ng/mL) (*p* = 0.042). The change in parathyroid (PTH) was statistically significantly different between the two groups (*p* = 0.031). In the Osteo-F group, the PTH significantly decreased after the experiment (34.58 ± 9.13 pg/mL) from the baseline (39.97 ± 10.99 pg/mL) (*p* = 0.001). The PTH/Ca ratio also confirmed a statistically significant difference between the two groups after 24 weeks (*p* = 0.028). Moreover, the ratio in the Osteo-F group significantly decreased (3.76 ± 0.99) compared with the baseline (4.38 ± 1.24) (*p* = 0.001).

In addition, the safety evaluation, including vital signs, e.g., blood pressure, temperature, and pulse, showed no significant changes, and no adverse events occurred in the subjects during the 24 weeks. 

## 4. Discussion

This study included pre-clinical studies using in vitro and in vivo models of postmenopausal osteoporosis, as well as clinical trials to confirm the positive effects of Osteo-F, a newly developed herbal formulation on osteoporosis/osteopenia based on the in silico prediction of the pharmacological effects and potential target pathway of Osteo-F on osteoporosis, with objectives for the experimental design and development of osteoporotic drugs with specific target profiles. 

We began with a computer-assisted network pharmacology analysis, which represents the virtual potential mechanism of a drug, with the objective of designing and developing osteoporotic drugs with specific target profiles [13]. A total of 90% of clinical trials fail in the process of drug discovery and development, even though the dose, efficacy, and toxicity of drug candidates are rigorously optimized at the pre-clinical stage [14]. In this regard, synchronizing pre-clinical results from in silico, in vitro, and in vivo studies is an effective and reliable approach to clinical trials [11]. From the beginning to estimating the therapeutic efficacy and underlying mechanisms of Osteo-F, a newly developed herbal formulation, we verified the correlation between Osteo-F and osteoporosis in the present study. A comparison of genes between Osteo-F and the summed osteoporosis sets demonstrated that 49.05% of the genes of Osteo-F were common between the Osteo-F and osteoporosis genes, indicating that Osteo-F might have a significant correlation with osteoporosis. We further conducted a functional enrichment analysis of the Osteo-F network to determine whether it had osteoanabolic effects of Osteo-F. The result showed that Osteo-F might affect the “regulation of osteoblast differentiation” and “osteoblast differentiation” via *GLI1*, *TWIST1*, *GJA1*, *BMP6*, *SMAD3*, etc., with high relevance scores. In addition, COL (*COL*), BMP-2 (*BMP-2*), and OCN (*BGLAP*) are included in the matched genes list for the GO process terms “regulation of osteoblast differentiation” and “osteoblast differentiation”. Through these results, Osteo-F was found to have a close connection with osteoporosis through the regulation of osteoblast differentiation shown by the network pharmacological analysis.

From those predicted results, we designed pre-clinical experiments to confirm the bone-forming property, thus providing convincing evidence for a clinical trial suitable for the amelioration of osteoporosis by the Osteo-F herbal formulation. One of the most important and widely used markers for osteoporosis is the level of BMD [15]. Based on the prediction via network pharmacology analysis, the level of BMD in the femoral bone of OVX-induced osteoporosis mice was significantly increased by Osteo-F. During the bone formation stage, osteoblasts, which secrete the bone organic matrix containing dense collagen layers, are differentiated and mineralized by secreting bone-forming molecules to contribute to the production of the mature bone matrix composite [16]. Under the pathological condition of dystrophic bone remodeling such as osteopenia and osteoporosis, additional stimulation to regenerate and form a new bone is required with an osteogenic treatment. For those reasons, the effects of Osteo-F on osteoblast osteogenic differentiation and mineralization were explored in differentiation medium-incubated SaOS-2 osteoblast-like cells. Osteo-F treatment considerably enhanced the mineralization of osteoblasts in SaOS-2 cells, and the mRNA expression of COL was significantly augmented in the presence of the osteoblast differentiation medium. These results indicated that Osteo-F could activate the differentiation and mineralization of osteoblast cells.

It is regarded positively, given the fact that the current therapeutic treatments for osteoporosis have focused on anti-resorptive drugs targeting the inhibition of osteoclasts maturation, proliferation, and activity for decades [17]. To date, the approved osteo-anabolic drugs by the Food and Drug Administration (FDA) are only Teriparatide (Forteo™), Abaloparatide (Tymlos™), and Romosozumab (Evenity™), while there are eight approved anti-resorptive medications including bisphosphonates. Thus, there is much attention on finding available anabolic treatments for osteoporosis. Considering the reality that it is difficult to not only slow bone loss but also increase bone density, the development of osteoblast-targeted drugs showing promising effects with fewer side effects is able to narrow the gap in the treatment of osteoporosis. 

In postmenopausal women, degenerative diseases such as osteoporosis and osteomalacia naturally progress with age, and bone density tends to continuously decrease [18]; thus, it is tough to obtain meaningful results on bone density increase in experimental and real-world settings [19]. Therefore, for bone health in this age group, the focus is on slowing bone loss rather than increasing bone density. The obtained results with the network analysis and the in vitro and in vitro data suggest that Osteo-F might have a bone-forming property, which has been reported in previous studies [8,9]. From these data, it was expected that the administration of Osteo-F would ameliorate the BMD level with the change in serum biomarkers related to bone formation in postmenopausal women in this study. In terms of the results of the bone density using DXA in the postmenopausal women of this study, when Oseto-F was supplemented for 24 weeks compared to the control group, the Z score of the lumbar spine increased. However, there was no significant difference in the BMD, BMC, and T-score. The Z-score is a value compared to the average bone density of the same age group [20]. It is used for diagnosis and treatment/intervention evaluation of all age groups, including menopausal and middle-aged women [21]. In the case of our participants, the Z-score is more suitable for comparing bone density in the same age group by targeting postmenopausal women [22]. These results confirm that Osteo-F has an improved effect on bone mineral density in postmenopausal women compared to the same age group.

Overall, the efficacy of Osteo-F as a treatment for osteoporosis has been proven through reliable data from osteopenia patients. In accordance with those results, the osteoanabolic mechanism of Osteo-F against osteoporosis was further authenticated. Various osteogenic-associated genes and molecules are responsible for the transcriptional mediator for the differentiation and maturation of osteoblasts. BSP-1 and OPN, bone-specific matrix genes, are considered key markers for producing the bone matrix [23]. BMP-2 is known to be a powerful osteosynthesis transcriptional mediator that controls bone development. It is also well known that BMP-2 transcription activates Runx2 and Osterix, contributing to the early stage of osteoblast differentiation from mesenchymal stem cells [24]. Runx2 expression induces the activation of Osterix, which clearly regulates the ALP and OCN expression, leading to osteoblastic differentiation [23]. Furthermore, ALP plays a bone turnover marker both in the early and late stages of the bone development process [15]. The expression of ALP has been reported to increase maximally during matrix maturation [25]. OCN, secreted from osteoblasts, is a predominant protein residing in the extracellular matrix and has been reported to be closely correlated with bone formation [26,27]. It is especially well established that OPN forms the OCN collagen complex that stretches and dissipates energy to inhibit bone cracks in situations where bones fracture [28]. In accordance with those reports, osteocalcin is regarded as a marker of osteoblast-derived bone formation at the maturation stage [29]. Thus, the effects of Osteo-F on osteoblast differentiation were confirmed by analyzing the expressions of bone-forming mediators in the differentiated and matured osteoblasts. Osteo-F treatment promoted the mRNA expressions of BSP-1, BMP-2, and OPN in differentiated osteoblast cells. Additionally, Runx2 and Osterix as early bone-forming proteins and OCN and ALP as mature osteogenesis proteins were significantly increased by Osteo-F. In particular, serum OCN levels were significantly increased in the Osteo-F orally administered osteoporotic mice, and the decreased OCN expressions by OVX were markedly increased in the femoral bone tissues by the Osteo-F treatment. The results revealed that the herbal formulation, Osteo-F, could promote a series of osteoblastic differentiations from BMP-2 to Runx2 and Osterix, ALP, and subsequently OCN. 

In a clinical trial to confirm the results of our pre-clinical study, supplementation with Osteo-F for 24 weeks resulted in a significant increase in the serum osteocalcin levels in postmenopausal women compared to the placebo. Osteocalcin, an osteogenic marker, helps predict the rate of bone loss and is used as an indicator of the rate of bone remodeling. Similar to our study, Filip et al. [30] found that postmenopausal female osteopenia subjects supplemented with 250 mg of olive extract and 1000 mg of Ca daily resulted in a significant increase in osteocalcin levels compared to the control group. In addition, serum PTH and serum PTH-to-Ca ratios decreased after Osteo-F supplementation, resulting in an increased bone mass and improved microstructure, leading to reduced fractures, indicating skeletal formation and recovery markers. To discuss these results in relation to the clinical data from osteopenia patients here, the roles of PTH/Ca in bone remodeling need to be described. In response to the serum Ca level, the pattern of PTH changes, showing catabolic and anabolic effects [31]. OCN expression is known to be regulated by PTH. Additionally, the functional activity of Runx2 could be regulated by PTH as well as BMP-2 [32]. It implied that the Osteo F supplement is an anabolic agent that increases bone formation. To discuss this, Betaine, known as a characteristic compound of *L. chinense*, stimulated the expressions of Runx2 and Osterix and induced the intracellular Ca level [33]. Additionally, it was reported that pinoresinol diglucoside, a main component of *E*. *ulmoides*, could enhance osteogenic differentiation with the activation of BMP-2, Runx2, Osterix, and ALP [34]. By contrast, the consisting components of *S. chinensis*, such as shizandrin A, schizandrin Bg, and schisantherin A, are regarded as effective inhibitors of osteoclastogenesis [35,36,37]. Although this study focused on the functional target of Osteo-F on osteoblastic bone formation through in silico analysis and clinical trials, we assumed that it could have synergetic effects on bone formation and bone resorption.

Considering these observations, it is concluded that there is a coordinated regulation of bone growth by Osteo-F, which is consistent with the predicted result from the network pharmacology analysis. A randomized, double-blind, placebo-controlled clinical trial showed that Osteo-F has anabolic effects on osteoporosis. Administration of Osteo-F for 24 weeks induced increases in the Z-score of the lumbar spine and T-score of the total femur with no adverse effects. Serum OCN, PTH, and PTH/Ca ratios were significantly increased and decreased, respectively, in the Osteo-F group compared to the placebo group. The anabolic properties of Osteo-F were confirmed in differentiated osteoblast cells, revealing that Osteo-F markedly execrated the formation of the extracellular matrix with an increase of Ca nodules. Osteogenic genes and proteins at the early and late stages, including COL (*COL*), BMP-2 (*BMP-2*), and OCN (*BGLAP*), were significantly increased by Osteo-F. The BMD level of the femur was significantly changed by Osteo-F in OVX-induced osteoporotic mice. OCN in the serum and femoral bone tissues was highly expressed by Osteo-F administration. Taken together, Osteo-F could efficaciously alleviate bone loss by promoting osteoblast-derived bone formation.

## 5. Conclusions

Drug development encompasses a connection between virtual drug candidate selection, pre-clinical studies, and clinical trials for successful “bench to bedside” development of drugs [38]. Upon integrating the experimental data including in silico, in vitro, in vivo, and clinical trials, this study provided reliable evidence that Osteo-F might be particularly relevant in bone-forming effects via activation of the differentiation and mineralization of osteoblasts in osteoporosis, demonstrating that Osteo-F is a novel bone anabolic treatment in postmenopausal women.

## Figures and Tables

**Table 1:** Bone mineral density and biochemical bone markers among Korean postmenopausal women at baseline and follow-up (24 weeks) of the intervention.

Variables	OSTEO-F (*n* = 39)				Placebo (*n* = 43)				*p*-Value ^‡^
	Baseline	Follow-Up	Change	*p*-Value ^†^	Baseline	Follow-Up	Change	*p*-Value ^†^	
Bone mineral density									
Lumbar spine									
BMD (g/cm^2^)	1.03 ± 0.13	1.03 ± 0.13	0.00 ± 0.02	0.572	1.02 ± 0.12	1.02 ± 0.12	−0.01 ± 0.02	0.103	0.113
BMC (g)	52.38 ± 11.31	52.41 ± 11.27	0.03 ±1.27	0.875	53.56 ± 11.57	53.33 ± 11.57	−0.23 ± 1.39	0.296	0.395
T-score	−0.95 ± 1.09	−0.93 ± 1.09	0.02 ± 0.19	0.633	−1.02 ± 1.00	−1.06 ± 1.00	−0.04 ± 0.18	0.153	0.164
Z-score	−0.23 ± 1.08	−0.16 ± 1.08	0.07 ± 0.20	0.045	−0.32 ± 0.96	−0.34 ± 0.97	−0.02 ± 0.19	0.544	0.048
Femur total									
BMD (g/cm^2^)	0.95 ± 0.09	0.95 ± 0.09	0.00 ± 0.01	0.063	0.95 ± 0.11	0.95 ± 0.11	0.00 ± 0.01	0.058	0.794
BMC (g)	28.07 ± 3.29	28.02 ± 3.35	−0.05 ± 0.50	0.555	28.29 ± 3.36	28.18 ± 3.34	−0.11 ± 0.44	0.103	0.538
T-score	−0.18 ± 0.76	−0.22 ± 0.78	−0.03 ± 0.09	0.044	−0.18 ± 0.96	−0.21 ± 0.95	−0.03 ± 0.12	0.096	0.988
Z-score	0.00 ± 0.78	0.02 ± 0.80	0.02 ± 0.12	0.438	0.00 ± 0.88	0.00 ± 0.88	0.00 ± 0.12	0.898	0.495
Biochemical bone markers									
OCN (ng/mL)	18.44± 6.14	19.23 ± 5.89	0.79 ± 3.02	0.110	21.41 ± 7.21	21.24 ±5.50	−0.10 ±2.58	0.813	0.042
PTH (pg/mL)	39.97 ± 10.99	34.58 ± 9.13	−5.39 ± 9.18	0.001	36.00 ± 10.61	35.30 ± 13.15	−0.70 ± 10.17	0.655	0.031
PTH/ Ca ratio	4.38 ± 1.24	3.76 ± 0.99	−0.62 ± 1.03	0.001	3.92 ± 1.23	3.85 ± 1.47	−0.07 ± 1.16	0.675	0.028

Values are expressed as means ± SD. ^†^ *p*-values were obtained by paired *t*-test within the group. ^‡^ Group differences of change were calculated using the general linear models (GLM) after adjusting for age. BMD: Bone mineral density, BMC: Bone mineral content, PTH: Parathyroid hormone.

## Data Availability

All data are available from the corresponding authors upon reasonable request.

## Supplementary Materials

The following supporting information can be downloaded at: https://www.mdpi.com/article/10.3390/cells12151992/s1, Table S1: Compounds of *Schizandra chinensis*, *Lycium chinense*, and *Eucommia ulmoides* from the TM-MC database; Table S2: Related genes of *Schizandra chinensis*, *Lycium chinense*, and *Eucommia ulmoides*; Table S3: Osteoporosis-related genes from GeneCards, MalaCards, and DisGeNet databases; Table S4: Osteoporosis-related genes in total.

## References

[1] Tu K.N., Lie J.D., Wan C.K.V., Cameron M., Austel A.G., Nguyen J.K., Van K., Hyun D. (2018). Osteoporosis: A Review of Treatment Options. Pharm. Ther..

[2] Siddiqui J.A., Partridge N.C. (2016). Physiological Bone Remodeling: Systemic Regulation and Growth Factor Involvement. Physiology.

[3] Black D.M., Rosen C.J. (2016). Postmenopausal Osteoporosis. N. Engl. J. Med..

[4] Anastasilakis A.D., Polyzos S.A., Makras P., Aubry-Rozier B., Kaouri S., Lamy O. (2017). Clinical Features of 24 Patients With Rebound-Associated Vertebral Fractures After Denosumab Discontinuation: Systematic Review and Additional Cases. J. Bone Min. Res..

[5] Jolette J., Attalla B., Varela A., Long G.G., Mellal N., Trimm S., Smith S.Y., Ominsky M.S., Hattersley G. (2017). Comparing the incidence of bone tumors in rats chronically exposed to the selective PTH type 1 receptor agonist abaloparatide or PTH(1-34). Regul. Toxicol. Pharmacol..

[6] Cosman F. (2020). Anabolic Therapy and Optimal Treatment Sequences for Patients With Osteoporosis at High Risk for Fracture. Endocr. Pract..

[7] Yoshida K., Oida H., Kobayashi T., Maruyama T., Tanaka M., Katayama T., Yamaguchi K., Segi E., Tsuboyama T., Matsushita M. (2002). Stimulation of bone formation and prevention of bone loss by prostaglandin E EP4 receptor activation. Proc. Natl. Acad. Sci. USA.

[8] Lee H., Kim M.H., Choi Y., Yang W.M. (2021). Ameliorative effects of Osteo-F, a newly developed herbal formula, on osteoporosis via activation of bone formation. J. Ethnopharmacol..

[9] Lee J.E., Kim M.H., Choi H., Yang W.M. (2017). Effects of Osteo-F, a new herbal formula, on osteoporosis via up-regulation of Runx2 and Osterix. RSC Adv..

[10] Hopkins A.L. (2008). Network pharmacology: The next paradigm in drug discovery. Nat. Chem. Biol..

[11] Abd-Algaleel S.A., Metwally A.A., Abdel-Bar H.M., Kassem D.H., Hathout R.M. (2021). Synchronizing In Silico, In Vitro, and In Vivo Studies for the Successful Nose to Brain Delivery of an Anticancer Molecule. Mol. Pharm..

[12] De Hao C., Xiao P.G. (2014). Network pharmacology: A Rosetta Stone for traditional Chinese medicine. Drug Dev. Res..

[13] Zhang R., Zhu X., Bai H., Ning K. (2019). Network Pharmacology Databases for Traditional Chinese Medicine: Review and Assessment. Front. Pharmacol..

[14] Duxin S., Wei G., Hongxiang H., Simon Z. (2022). Why 90% of clinical drug development fails and how to improve it?. Acta Pharm. Sin. B.

[15] Kuo T.R., Chen C.H. (2017). Bone biomarker for the clinical assessment of osteoporosis: Recent developments and future perspectives. Biomark. Res..

[16] Blair H.C., Larrouture Q.C., Li Y., Lin H., Beer-Stoltz D., Liu L., Tuan R.S., Robinson L.J., Schlesinger P.H., Nelson D.J. (2017). Osteoblast Differentiation and Bone Matrix Formation In Vivo and In Vitro. Tissue Eng. Part. B Rev..

[17] Corrado A., Sanpaolo E.R., Di Bello S., Cantatore F.P. (2017). Osteoblast as a target of anti-osteoporotic treatment. Postgrad. Med..

[18] Ji M.X., Yu Q. (2015). Primary osteoporosis in postmenopausal women. Chronic Dis. Transl. Med..

[19] Tanphiriyakun T., Rojanasthien S., Khumrin P. (2021). Bone mineral density response prediction following osteoporosis treatment using machine learning to aid personalized therapy. Sci. Rep..

[20] Wendlova J. (2002). Differences in distribution of T-scores and Z-scores among bone densitometry tests in postmenopausal women (a comparative study). Wien. Med. Wochenschr..

[21] Shepherd J.A., Blake G.M. (2007). T-scores and Z-scores. J. Clin. Densitom..

[22] Licata A.A. (2006). Diagnosing primary osteoporosis: It’s more than a T score. Cleve Clin. J. Med..

[23] Matsubara T., Kida K., Yamaguchi A., Hata K., Ichida F., Meguro H., Aburatani H., Nishimura R., Yoneda T. (2008). BMP2 regulates Osterix through Msx2 and Runx2 during osteoblast differentiation. J. Biol. Chem..

[24] Chen D., Harris M.A., Rossini G., Dunstan C.R., Dallas S.L., Feng J.Q., Mundy G.R., Harris S.E. (1997). Bone morphogenetic protein 2 (BMP-2) enhances BMP-3, BMP-4, and bone cell differentiation marker gene expression during the induction of mineralized bone matrix formation in cultures of fetal rat calvarial osteoblasts. Calcif. Tissue Int..

[25] Siggelkow H., Rebenstorff K., Kurre W., Niedhart C., Engel I., Schulz H., Atkinson M.J., Hufner M. (1999). Development of the osteoblast phenotype in primary human osteoblasts in culture: Comparison with rat calvarial cells in osteoblast differentiation. J. Cell Biochem..

[26] Manolagas S.C. (2020). Osteocalcin promotes bone mineralization but is not a hormone. PLoS Genet..

[27] Delmas P.D., Eastell R., Garnero P., Seibel M.J., Stepan J., Committee of Scientific Advisors of the International Osteoporosis Foundation (2000). The use of biochemical markers of bone turnover in osteoporosis. Osteoporos. Int..

[28] Nikel O., Laurencin D., McCallum S.A., Gundberg C.M., Vashishth D. (2013). NMR investigation of the role of osteocalcin and osteopontin at the organic-inorganic interface in bone. Langmuir.

[29] De Toni L., Jawich K., De Rocco Ponce M., Di Nisio A., Foresta C. (2020). Osteocalcin: A Protein Hormone Connecting Metabolism, Bone and Testis Function. Protein Pept. Lett..

[30] Filip R., Possemiers S., Heyerick A., Pinheiro I., Raszewski G., Davicco M.J., Coxam V. (2015). Twelve-month consumption of a polyphenol extract from olive (*Olea europaea*) in a double blind, randomized trial increases serum total osteocalcin levels and improves serum lipid profiles in postmenopausal women with osteopenia. J. Nutr. Health Aging.

[31] Jiang D., Franceschi R.T., Boules H., Xiao G. (2004). Parathyroid hormone induction of the osteocalcin gene. Requirement for an osteoblast-specific element 1 sequence in the promoter and involvement of multiple-signaling pathways. J. Biol. Chem..

[32] Yang S., Wei D., Wang D., Phimphilai M., Krebsbach P.H., Franceschi R.T. (2003). In vitro and in vivo synergistic interactions between the Runx2/Cbfa1 transcription factor and bone morphogenetic protein-2 in stimulating osteoblast differentiation. J. Bone Min. Res..

[33] Villa I., Senesi P., Montesano A., Ferraretto A., Vacante F., Spinello A., Bottani M., Bolamperti S., Rubinacci A., Luzi L. (2017). Betaine promotes cell differentiation of human osteoblasts in primary culture. J. Transl. Med..

[34] Zhang N., Xie H., Wu Y., Han Y., Wang X. (2020). Pinoresinol Diglucoside Relieves Osteoporosis Through Enhancing Osteogenic Differentiation via Activating Phosphatidylinositol-3-Kinase/Protein Kinase B Signaling Pathway. J. Biomater. Tissue Eng..

[35] Ni S., Qian Z., Yuan Y., Li D., Zhong Z., Ghorbani F., Zhang X., Zhang F., Zhang Z., Liu Z. (2020). Schisandrin A restrains osteoclastogenesis by inhibiting reactive oxygen species and activating Nrf2 signalling. Cell Prolif..

[36] Wang J., Fang Z., Song C., Kang H., Guo Q., Dong Y., Zhang Y., Peng R., Guan H., Li F. (2020). Schisandrin B Inhibits Osteoclastogenesis and Protects Against Ovariectomy-Induced Bone Loss. Front. Pharmacol..

[37] He Y., Zhang Q., Shen Y., Chen X., Zhou F., Peng D. (2014). Schisantherin A suppresses osteoclast formation and wear particle-induced osteolysis via modulating RANKL signaling pathways. Biochem. Biophys. Res. Commun..

[38] Steinmetz K.L., Spack E.G. (2009). The basics of preclinical drug development for neurodegenerative disease indications. BMC Neurol..

